# Uterine Prolapse Across the Female Lifespan: Clinical Insights and Practical Considerations from Greece

**DOI:** 10.3390/nursrep15060212

**Published:** 2025-06-12

**Authors:** Athina Loukopoulou, Eleni Tzanni, Anastasia Bothou, Evdokia Billis, Christina Nanou, Giannoula Kyrkou, Victoria Vivilaki, Anna Deltsidou

**Affiliations:** 1 Health Center of Atalanti, 35200 Atalanti, Greece; athenaloukopoulou@yahoo.gr; 2 Health Center of Lavrio, 19500 Athens, Greece; 3 Department of Midwifery, University of West Attica, 12243 Athens, Greece; nanouxv@uniwa.gr (C.N.); ikirkou@uniwa.gr (G.K.); vvivilaki@uniwa.gr (V.V.); adeltsidou@uniwa.gr (A.D.); 4 Department of Physiotherapy, University of Patras, Rio, Achaia, 26504 Patra, Greece; billis@upatras.gr

**Keywords:** Australian Pelvic Floor Questionnaire (APFQ), nursing, Pelvic Organ Prolapse Quantification (POP-Q) system, pelvic organ prolapse, prevalence, risk factors, uterine prolapse

## Abstract

**Objective:** The aim of this study is to investigate uterine prolapse (UP) among women attending a semi-urban health center for routine gynecological examinations. Specifically, the study explores the potential association between UP and various established or suspected risk factors, including age, menopausal status, number and mode of deliveries, birth weight, smoking habits, and body mass index (BMI). Furthermore, it examines the relationship between the presence or severity of UP and the scores of specific questionnaires and their subscales. Finally, the study seeks to develop a predictive model for the likelihood of UP based on questionnaire responses. **Methods:** A quantitative study was conducted at the gynecological department of a health center in Greece from January 2021 to October 2022. A total of 134 women were recruited using convenience sampling during routine gynecological visits. The degree of prolapse was classified according to the International Continence Society (ICS) Pelvic Organ Prolapse Quantification (POP-Q) classification system. Data collection also included the use of validated instruments: the Australian Pelvic Floor Questionnaire (APFQ), the Urogenital Distress Inventory-6 (UDI-6), the Pelvic Floor Distress Inventory-20 (PFDI-20), and the Pelvic Floor Impact Questionnaire-7 (PFIQ-7). The data were processed with the Statistical Package for the Social Sciences (SPSS) v25. **Results:** Of the 134 participants, 21 (15.7%) aged 21 to 82 showed signs of UP, while 113 women (84.3%) did not. The average age of the women with UP was 55 years. Fourteen (10.4%) of these women were diagnosed with UP stage I, three of them (2.2%) with stage II, and four of them (3%) with stage III UP. There were no stage IV UP incidents. The risk factors associated with the disease include age, mode of delivery, parity, and duration of menopause. Regarding parity, every subsequent birth after the first one increases the likelihood of a UP incident by approximately 125%. **Conclusions:** Most women with UP did not exhibit severe symptoms, as UP typically does not manifest symptoms until it reaches a final stage. Considering the population aging and the increase in morbidity, a regular pelvic organ prolapse (POP) checkup should be established to facilitate early recognition, prevention, and treatment of symptoms. This study offers a potential tool for non-invasive screening to facilitate identifying UP in women early, which has not been previously reported.

## 1. Introduction

The term “uterine prolapse” (UP) describes the descent of the uterus and cervix into the vagina [1,2]. Mild prolapse frequently has no symptoms [2]. However, moderate and severe prolapse can result in vaginal swelling and pressure, sexual dysfunction [3], and symptoms related to the bladder and intestine [2,4,5]. Higher levels of discomfort brought on by prolapse symptoms are linked to lower quality of life (QOL) [6]. The etiology of pelvic organ prolapse (POP) is multifaceted and complex [4,7]. The risk variables that are most frequently mentioned are obstetric or family history, age, and menopause [5]. Treatment options to support UP include physiotherapy, pessaries, and surgery [4].

POP is generally determined by clinical examination using the International Society for POP Classification (ICS POP-Q) system [7]. It is a useful tool that is recommended by major international health organizations to objectively assess and document the degree of prolapse, to enhance communication between clinicians and researchers, to objectively monitor changes in a patient over time, and to evaluate the success and durability of various surgical and non-surgical treatments [3].

It is difficult to determine exactly the number of women who are affected by UP because sometimes they have no symptoms [5], while those with symptoms sometimes do not seek medical care. The prevalence of UP based on reported symptoms is much lower (3–6%) than the prevalence detected at clinical examination (41–50%) [4]. It is estimated that 40–50% of women aged > 45 years experience some degree of POP during their lifetime [8], a proportion that will continue to increase due to the aging populations in developed countries [7]. The risk of a woman undergoing surgery for POP by the age of 79 years is 11–19%, and about 1/3 of these women will require reoperation due to recurrence [8,9,10,11].

The occurrence of UP is multifactorial in etiology [12]. Anatomical, genetic, physiological, lifestyle, and reproductive factors interact during a woman’s lifetime, and the combination of these can result in some degree of prolapse [12,13]. Risk factors can be divided into modifiable and non-modifiable [14]. Non-modifiable potential risk factors can include age, white race, menopause (≥10 years) [5], chronic obstructive lung disease, bone abnormalities, personal and family history of genital prolapse, pelvic surgery, chronic constipation, congenital or acquired connective tissue abnormalities, pelvic floor (PF) denervation or weakness, and factors associated with chronically elevated intra-abdominal pressure [15]. While modifiable potential risk factors include body weight, obesity, vaginal delivery, multiple deliveries [11], instrumental delivery [16], smoking, fetal macrosomia, perineal rupture, occupation, and low socioeconomic status [13].

In Greece, epidemiological data on UP are limited, particularly in semi-urban or rural populations where access to specialized gynecological services may be restricted. Early stages of prolapse often go unrecognized or unreported in such settings. Therefore, research focusing on these underrepresented populations is essential to support early diagnosis and inform public health strategies.

This study investigates UP among women attending a semi-urban health center in Greece for routine gynecological examinations. Specifically, it explores associations between UP and established or suspected risk factors, including age, menopausal status, number and mode of deliveries, birth weight, smoking habits, and body mass index (BMI). Furthermore, it examines the relationship between the presence or severity of UP and the scores of validated pelvic floor questionnaires and their subscales (APFQ, UDI-6, PFDI-20, PFIQ-7). Finally, the study aims to develop a predictive model for the likelihood of UP based on questionnaire responses. This is the first study to offer a potential tool for non-invasive screening to facilitate identifying women with UP early, which has not been previously reported.

## 2. Materials and Methods

In this observational, cross-sectional, analytical study, an attempt was made to classify the degree of UP and to investigate, record, and evaluate the symptoms of UP and their impact on women’s QOL.

The main study’s objectives were to explore the following questions: Is there an association of UP with risk factors such as age, menopause, number of deliveries, birth weight, mode of delivery, smoking, and body mass index (BMI)?

Is there a correlation between UP and the scores on the questionnaires and their subscales?

Is it possible to create a predictive model for the probability of UP using the named questionnaires?

### 2.1. Study’s Sample—Ethical Considerations

One hundred thirty-four women attending the gynecological–obstetric clinic of a health center in Greece for regular gynecological checkup participated in the study. The women were fully informed about the purpose of the study and voluntarily agreed to be evaluated for possible UP. The degree of UP was classified according to the ICS POP-Q. They then agreed to complete the PF questionnaire, which was preceded by the participants’ written consent according to the Declaration of Helsinki, as revised in 2013. The study was approved by the Research Ethics Committee of the University of West Attica, Athens, Greece, under protocol number 71514/06-10-2020. The sample size was determined based on feasibility and consistency with similar studies, and formal power analysis was performed.

Inclusion criteria were as follows: (1) female sex, (2) age ≥ 18 years, (3) ability to communicate in Greek, and (4) attending a routine gynecological examination.

Exclusion criteria included the following: (1) current pregnancy, (2) history of hysterectomy or pelvic malignancy, and (3) cognitive impairment interfering with questionnaire completion.

### 2.2. PF Questionnaires and Statistical Analysis

A total of three questionnaires were used, namely:

The Australian PF Questionnaire (APFQ)
The PF Distress Inventory/PF Disability Index (PFDI-20)
The PF Impact Questionnaire-7 (PFIQ-7) for the three different systems (urinary, gastrointestinal, and reproductive).
The personal data form was developed following an in-depth literature review and was evaluated for content validity by two experienced gynecologists.

The questionnaire also included demographic questions about factors that have been associated with UP, such as age, weight, BMI, number of deliveries, birth weight, smoking, serious medical conditions, and medication.

More specifically, the APFQ also known as the Queensland PF questionnaire, which has been applied to numerous populations as in Greece [17], consists of 42 questions, most on a Likert-type scale, divided into four sections [18]: bladder function (questions 1–15, maximum score of 45), bowel function (questions 16–27, maximum score of 34), prolapse symptoms (questions 28–32, maximum score of 15), and sexual function (questions 33–42, maximum score of 21), all scaled up to a 10-point scale. Based on these scores, health professionals can judge whether there is a risk of PF prolapse and to what extent, without requiring a prior examination of the patient. The higher the scores on these subscales, as well as on the overall PFQ scale, the more severe the symptoms experienced by patients.

The PDFI-20 questionnaire consists of 20 questions, and its strength is that it is a short form that gives a comprehensive assessment of the impact of PF disorders on women’s QOL. In this study, only the subscale of bladder-related questions, the Urinary Distress Inventory-6 (UDI-6) consisting of 6 Likert-type scale questions, was used. The maximum score is 133.3 [19].

The PFIQ-7 questionnaire [20] consisted of 7 Likert-type questions in each subscale, about the impact on women’s daily life related to bladder problems (Urinary Impact Questionnaire UIQ-7), bowel and/or rectum (Colorectal–Anal Impact Questionnaire-7 CRAIQ-7), and vagina or pelvis (POP Impact Questionnaire POPIQ-7). This is a health-related quality of life questionnaire for women with PF disorders. The maximum score of each subscale is 133.3 and the total score is 400 [21]. Statistical analysis was conducted using the Statistical Package for the Social Sciences (SPSS), version 25. Independent-samples t-tests and ANOVA were used to compare questionnaire scores across prolapse stages. Pearson’s correlation was applied to assess relationships between numerical variables. A *p*-value < 0.05 was considered statistically significant.

### 2.3. Reliability of Scales

Cronbach’s Alpha was performed to measure the internal consistency of the subscales and scales in the study. Of the APFQ subscales, PFQ-Bladder (0.841) had good reliability while the remaining three, PFQ-Bowel (0.798), PFQ-POP (0.783), and PFQ-Sexual function (0.734), had acceptable reliability. The PFDI/UDI-6 Total (0.834) had good reliability. The PFIQ-7/UIQ-7-Bladder (0.958), PFIQ-7/UIQ-7-Bowel (0.965), and PFIQ-7/UIQ-7-Vaginal (0.958) subscales and the PFIQ-7/UIQ-7 total score (0.966) had very high reliability.

### 2.4. Study’s Limitations

This study has certain limitations. The sample size and the number of UP cases were relatively small, which may have limited the strength of some associations and the predictive model. As a cross-sectional study, it cannot establish causality. The use of self-reported questionnaires may also carry a risk of reporting bias. Lastly, the absence of advanced UP stages and the single-center setting may affect generalizability. Despite these limitations, the study provides useful insights for primary care and highlights the value of non-invasive screening tools.

## 3. Results

The sample consisted of 134 women aged between 21 and 82 years. The mean age was 51 years and the mean value of BMI was 26.51.

Of the 134 women, 75 (56%) were menopausal and the minimum value of their last period was 0.6 years, the maximum was 39 years, and the mean value was 11.21 years ago (Table 1).

In addition, 9.7% of the sample reported urinary incontinence symptoms as the main problem and reason for their visit, 4.5% reported prolapse, 1.5% reported gynecological cancer, and 5.2% reported other various conditions.

All the women in the sample were classified based on the POP-Q system of quantifying POP during clinical examination and 21 (15.7%) had some degree of UP compared to 113 (84.3%). Of the women with UP, 14 (10.4%) had stage I prolapse, 3 (2.2%) had stage II prolapse, and 4 (3%) had stage III prolapse. No women were found with stage IV UP.

### 3.1. Obstetric History

Of the women, 20 (14.9%) did not have a child, 16 (11.9%) had one child, 70 (52.2%) had two children, 22 (16.4%) had three children, 5 (3.7%) had four children, and 1 (0.7%) had five or more children. Among the women who had at least one child, the first delivery in 73 cases (64%) was normal, in 4 cases (3.5%) it occurred by vacuum birth, in 3 cases (2.6%) it occurred by forceps, in 24 cases (21.1%) it occurred by planned cesarean section (CS), and in 10 cases (8.8%) it occurred by emergency CS.

### 3.2. Age and UP

Spearman’s rho coefficient was applied to determine a correlation between age and UP. Thus, among women up to 50 years of age, 12.9% were diagnosed with UP, while 87.1% did not. In the age group above 50 years, 18.1% of women were diagnosed with UP compared to 81.9% of those who had not. The risk ratio was 18.1/12.9 = 1.40. Also, out of all women diagnosed with UP, 61.9% are over 50 compared to 38.1% who are under 50. Therefore, age has an important role in the incidence of UP.

### 3.3. Number of Births and UP

According to our results, the number of deliveries affects the risk of UP (*p* < 0.005). Cases with three or more deliveries were then grouped into a category and it was found that, out of a total of 19 women who had no deliveries, only 1 (5.3%) had UP. Of the 16 women who had one delivery, one (6.3%) had UP. Of the 71 women with two deliveries, 10 (14.1%) had UP. Finally, of the 28 women with ≥3 deliveries, 9 women (32.1%) had symptoms of UP.

Therefore, there was a continuous increase in the percentage (within each category) of women who had experienced UP. The odds ratios per two of the consecutive categories of number of deliveries are as follows:

Between one and no births: 6.3/5.3 = 1.19
Between two and one birth: 14.1/6.3 = 2.24
Between three or more and two births: 32.1/14.1 = 2.28

Thus, after the first delivery, the probability of developing UP increases by 19%; after the second delivery, the probability of developing UP increases by 124%; after the third delivery, the likelihood of developing UP increases by 128% (Table 2).

### 3.4. Delivery Mode and UP

The X^2^ test showed a *p*-value = 0.015 < 0.05, so the null hypothesis, that there is no correlation between mode of delivery and uterine prolapse, is rejected (Table 2).

### 3.5. Menopause and UP

Of the sample, seventy-five women (56%) had entered menopause. Of these, 12 (16%) had been diagnosed with UP and 63 (84%) had not. In contrast, 59 women (44%) had entered menopause. Of these, 9 (15.3% of all menopausal women) had been diagnosed with UP, and 50 (84.7%) had not. Our study did not find a correlation between menopause and UP. However, studying the subset of menopausal women and examining the correlation between the duration of menopause and UP showed a statistically significant correlation (*p* = 0.008), which was moderate and positive (rho, *p* = 0.303).

### 3.6. Model for Predicting the Probability of UP

Logistic regression was applied with the dependent variable Pop_Q_nomimal as the dichotomous variable, which takes a value of zero (0) if a woman does not have UP and a value of one (1) if they do. Independent variables were considered the scores of the subscales in each questionnaire. The backward stepwise method was used, in which all variables are entered at the beginning, and the least statistically significant is removed, with a threshold of *p*-value = 0.010, until all variables remaining in the model do not exceed this limit. One variable, PFQ-Bowel, was removed, leaving the remaining three: PFQ-Bladder, PFQ-POP, and PFQ-Sexual Function (Table 3).

The prediction model is as follows:

$$\ln\left(\frac{p}{1-p}\right)=-2.434+\;0.902\;*\;PFQ_{Bladder}+0.674\;*\;PFQ_{POP}-0.473\;*\;PFQ_{sexualFunction}$$

where $ln(\frac{p}{1-p})$ is the natural logarithm of the odds ratio, with p being the probability that a woman will have UP and 1 − *p* being the probability that she will not. If the above logarithm is negative, then the probability that a woman will have UP is less than the probability that she will not. If it is positive, the probability of not having UP is less than the probability of having it; finally, if the logarithm of the odds ratio is zero then the two probabilities are equal.

The constant −2.434 indicates that, if all independent variables take the value zero (0), then the logarithm of the probabilities will be equal to −2.434, or the odds ratio will be $\frac{p}{1-p}=e^{-2.434}=0.088$ or 8.8%.

Increasing the score in PFQ_Bladder increases the log-likelihood ratio by 0.902 points or, namely, increases the likelihood ratio by 2.465 times.

Increasing the score in PFQ_POP increases the logarithm of probability by 0.674 units or, namely, increases the likelihood ratio by 1.962 times.

Increasing the score on PFQ_Sexual Function decreases the reasonable probability by 0.473 points or, otherwise, decreases the odds ratio by 0.623 times.

The model was applied to our sample and was able to be verified in 88.3% of the cases, as shown in Table 4.

## 4. Discussion

UP is one of the gynecological morbidities of concern in the female population [22] and causes significant physical and emotional distress [13].

In the present study, 134 women, with a mean age of 51.51 years (range: 21–82 years), were studied. In southwest Nigeria, the mean age was 51.4 years, while studies in India ranged from 50.1 to 52.8 years [22,23].

The prevalence of UP cannot be clearly estimated [24,25]. Significant differences in its variation between studies appear, even if they are from the same country [Ethiopia [1], Ghana [24], Saudi Arabia [26]]. This is because prevalence can be affected by sociocultural, occupational, and racial characteristics, and by the behavior of people seeking healthcare [1,22,24,27]. Moreover, differences in definitions of prevalence, the inclusion of different age groups, and methods of diagnosis and classification may play a role [1,27].

The prevalence of UP starts from 0.07% (South Korea) [25] or 1% (Northern and Eastern Ethiopia) [1,28] and reaches >64.6% in a study from Tanzania. Increased prevalence rates have been reported in studies from Gambia, West Africa (46%), Lebanon (49.6%), and Iran (53.6%) [14]. In a study conducted in Southwest Ethiopia, the rate was 22.3% [28]. In Eastern Ethiopia, the prevalence of UP was 9.5%, which is relatively similar to that of this study and that of studies from Nepal (13%) and Southern Ghana (12.07%) [1,24]. In the Women’s Health Initiative (WHI) study, conducted among 16,616 women aged 50 to 79 years, the rate of UP was 14.2% [29].

UP is of multifactorial etiology. In the present study, age, mode of delivery, number of deliveries, and duration of menopause were found to be associated with UP.

The results of our study are consistent with those of a systematic review and meta-analysis [14]. The data from this study showed that age, BMI, the birth weight of the child, and damage to the anal sphincter muscle, mainly during delivery, were statistically significant risk factors, while CS and smoking were protective factors for primary prolapse. The length of the urogenital septum and the number and mode of deliveries are identified as confirmed risk factors [14]. A strongly significant risk factor is delivery with forceps, compared to vacuum birth, which did not show a significant association [13,14,30]. In the present study, CS reduces the probability of UP.

A retrospective study showed almost the same results for factors associated with genital prolapse [31]. BMI, age, number, and/or mode of delivery are the main factors reported in studies from the USA [32], Australia [33], Sweden [34], Bangladesh [35], and Saudi Arabia [26,27]. Birth weight, menopause, history of pelvic surgery (hysterectomy) [32], ethnicity [27], and perineal tears [31] were added to the list of factors of genital prolapse, along with prolonged labor, lasting >24 h, and agricultural occupation, as showed in a study from Uganda [36].

Occupation, especially manual work, involving strain or lifting heavy objects, is presented as a risk factor in a study from Ghana [24]. The prevalence of POP in this study was 2.68%, with 80.5% of this being UP and cystocele [24]. Similar results were found in the South Korean study, with UP found in 49.9% of the total cases of POP, followed by cystocele [25], in contrast with a study from Saudi Arabia with POP, including UP, showing lower detection rates compared to bladder and bowel dysfunction [27].

The high number of deliveries and the lesions that occur during the first delivery are factors that may aggravate UP. Thus, the first birth is more likely to cause the greatest damage to the PF by 96% [37]. According to our study, the probability of UP increases by 128% after the third delivery and after the 2nd by 124%. The results of our study are similar to those of a study from India which reported that women who had undergone three deliveries had four times more risk of UP and women with more than or equal to four deliveries had eight times greater risk of UP [22]. However, in these studies from developing countries, only a small percentage of deliveries were conducted in health facilities (Ethiopia 28.9%, India 13.9%) [22]. Thus, in these studies, the place of delivery, the person from whom the delivery is performed, the age at first delivery, the history of abortion, and the heavy work during the postnatal period emerged as risk factors [23,28].

In our study, the participating women reported urinary tract symptoms, but when the stage of prolapse increased (stage III), they also reported symptoms of prolapse. The main symptoms, showing moderate–almost strong correlation, are the feeling of a lump or protruding part from the vagina. Similar results are reported in studies from Nigeria [38] and Indonesia [39].

The symptoms of UP and POP, often in the early stages, have little effect on women’s QOL and are temporarily tolerable [40]. However, if they persist or worsen, they can significantly affect women’s health and social wellbeing [40]. The QOL of women studied in this study showed a low correlation with bladder symptoms and prolapse. This may be due to the early stages of UP. However, in countries such as India, Ethiopia, and Nepal, cultural circumstances may impede women from making decisions about their own healthcare, meaning they may be reluctant to visit health facilities, even in advanced stages of prolapse, to discuss their condition, as they may feel fear or shame [41].

The questionnaires used in this study, the APFQ, the UDI-6 subscale from the PFDI-20, and the PFIQ-7 are reliable and have been used in studies in Saudi Arabia [26,27,42].

## 5. Conclusions

The number of women with POP problems is expected to increase in the coming years due to the aging population. This study provides new insights into identifying UP in a semi-urban primary care setting using validated pelvic floor questionnaires. The findings highlight the clinical relevance of routine screening and the role of primary care professionals in recognizing the early signs and symptoms of UP.

Importantly, a predictive model was developed based on questionnaire responses, offering a potential tool for early, non-invasive risk stratification in everyday practice. This approach could support timely referrals and reduce the underdiagnosis of UP in underserved populations.

Further research with larger, more diverse samples and longitudinal designs is needed to externally validate the predictive model and explore its integration into clinical workflows and public health strategies.

## Figures and Tables

**Table 1:** Descriptive characteristics of the study participants (N = 134).

Variable	Mean	Median	Standard Deviation (s)	Minimum	Maximum
Age (years)	51.51	52	13.181	21	82
Height (cm)	163.39	163.5	6.421	149	183
Weight (kg)	70.694	69	12.223	48	109
Body Mass Index (BMI)	26.5132	25.6203	4.5133	18.29	40.86
Years Since Last Menstrual Period	11.2147	10	8.2454	0.6	39
Duration of Problem (months)	83.22	60	92.865	1	420

**Table 2:** Percentages (relative frequencies) of UP in relation to the number of deliveries of study participants and X^2^ test for the correlation between mode of delivery and UP.

				POP_Q_nomimal		Total
				0.00	1.00	
Number of deliveries	0.00		Count	18	1	19
			% within births2	94.7%	5.3%	100.0%
			% within POP_Q_nomimal	15.9%	4.8%	14.2%
			% of Total	13.4%	0.7%	14.2%
	1.00		Count	15	1	16
			% within births2	93.8%	6.3%	100.0%
			% within POP_Q_nomimal	13.3%	4.8%	11.9%
			% of Total	11.2%	0.7%	11.9%
	2.00		Count	61	10	71
			% within births2	85.9%	14.1%	100.0%
			% within POP_Q_nomimal	54.0%	47.6%	53.0%
			% of Total	45.5%	7.5%	53.0%
	3.00		Count	19	9	28
			% within births2	67.9%	32.1%	100.0%
			% within POP_Q_nomimal	16.8%	42.9%	20.9%
			% of Total	14.2%	6.7%	20.9%
Total		Count		113	21	134
		% within births2		84.3%	15.7%	100.0%
		% within POP_Q_nomimal		100.0%	100.0%	100.0%
		% of Total		84.3%	15.7%	100.0%
Mode of delivery	0.00	Count		59	17	76
		% within birth type		77.6%	22.4%	100.0%
		% within POP_Q_nomimal		52.2%	81.0%	56.7%
		% of Total		44.0%	12.7%	56.7%
	1.00	Count		54	4	58
		% within birth type		93.1%	6.9%	100.0%
		% within POP_Q_nomimal		47.8%	19.0%	43.3%
		% of Total		40.3%	3.0%	43.3%
Total		Count		113	21	134
		% within birth type		84.3%	15.7%	100.0%
		% within POP_Q_nomimal		100.0%	100.0%	100.0%
		% of Total		84.3%	15.7%	100.0%

**Table 3:** Logistic regression with the dichotomous variable Pop_Q_nomimal as the dependent variable and the scores of the subscales in each questionnaire as independent variables.

		B	S.E.	Wald	df	Sig.	Exp(B)
Step 2 ^a^	PFQ-Bladder subscore (1–15)	0.902	0.271	11.086	1	0.001	2.465
	PFQ-POP subscore (28–32)	0.674	0.303	4.957	1	0.026	1.962
	PFQ-Sexual function subscore (33–42)	−0.473	0.252	3.525	1	0.060	0.623
	Constant	−2.434	0.474	26.327	1	0.000	0.088

^a^ Variable(s) entered on step 1: PFQ-Bladder (1–15), PFQ-Bowel subscore (16–27), PFQ-POP subscore (28–32), PFQ-Sexual function subscore (33–42), B: Regression coefficient, S.E.: Standard Error, Wald: Wald test statistic, df: Degrees of Freedom, Sig.: Significance (*p*-value), Exp(B): Exponentiated B (odds ratio).

**Table 4:** Model for predicting the probability of UP.

Classification Table ^a^					
Observed		Predicted			
		POP_Q_nomimal			Percentage Correct
		0.00		1.00	
Step 1	POP_Q_nomimal	0.00	99	2	98.0
		1.00	12	7	36.8
	Overall Percentage				88.3
Step 2	POP_Q_nomimal	0.00	100	1	99.0
		1.00	13	6	31.6
	Overall Percentage				88.3

^a^ The cut value is 0.500.

## Data Availability

The data that support the findings of this study are available from the corresponding author upon reasonable request.

## Public Involvement Statement

No patients or members of the public were directly involved in the design, conduct, reporting, or dissemination plans of this research.

## Guidelines and Standards Statement

This study follows the STROBE guidelines for observational studies.

## References

[1] Badacho A.S., Lelu M.A., Gelan Z., Woltamo D.D. (2022). Uterine prolapse and associated factors among reproductive-age women in south-west Ethiopia: A community-based cross-sectional study. PLoS ONE.

[2] Hemming C., Constable L., Goulao B., Kilonzo M., Boyers D., Elders A., Cooper K., Smith A., Freeman R., Breeman S. (2020). Surgical interventions for uterine prolapse and for vault prolapse: The two VUE RCTs. Health Technol. Assess..

[3] American College of Obstetricians and Gynecologists (ACOG) (2017). Practice Bulletin. Pelvic Organ Prolapse. Am. Coll. Obstet. Gynecol..

[4] Espiño-Albela A., Castaño-García C., Díaz-Mohedo E., Ibáñez-Vera A.J. (2022). Effects of pelvic-floor muscle training in patients with pelvic organ prolapse approached with surgery vs. conservative treatment: A systematic review. J. Pers. Med..

[5] Rountis A., Zacharakis D., Athanasiou S., Kathopoulis N., Grigoriadis T., Rountis A.N. (2021). The role of laparoscopic surgery in the treatment of advanced uterine prolapse: A systematic review of the literature. Cureus.

[6] Fontenele M.Q.S., Moreira M.A., de Moura A.C.R., de Figueiredo V.B., Driusso P., Nascimento S.L. (2021). Pelvic floor dysfunction distress is correlated with quality of life, but not with muscle function. Arch. Gynecol. Obstet..

[7] Shek K.L., Dietz H.P. (2016). Assessment of pelvic organ prolapse: A review. Ultrasound Obstet. Gynecol..

[8] Enklaar R.A., Knapen F.M., Schulten S.F., van Osch L.A., van Leijsen S.A., Gondrie E.T., Weemhoff M. (2023). The modified Manchester Fothergill procedure compared with vaginal hysterectomy with low uterosacral ligament suspension in patients with pelvic organ prolapse: Long-term outcome. Int. Urogynecol. J..

[9] Scime N.V., Ramage K., Brennand E.A. (2021). Protocol for a prospective multisite cohort study investigating hysterectomy versus uterine preservation for pelvic organ prolapse surgery: The HUPPS study. BMJ Open.

[10] Brunes M., Johannesson U., Drca A., Bergman I., Söderberg M., Warnqvist A., Ek M. (2022). Recurrent surgery in uterine prolapse: A nationwide register study. Acta Obstet. Gynecol. Scandinavica.

[11] Shi W., Guo L. (2023). Risk factors for the recurrence of pelvic organ prolapse: A meta-analysis. J. Obstet. Gynaecol..

[12] Aboseif C., Liu P. (2024). Pelvic Organ Prolapse. StatPearls [Internet].

[13] Obsa M.S., Worji T., Kedir N., Kute N. (2022). Risk factors of pelvic organ prolapse at Asella Teaching and Referral Hospital: Unmatched case control study. Front. Glob. Womens Health.

[14] Schulten S.F., Claas-Quax M.J., Weemhoff M., van Eijndhoven H.W., van Leijsen S.A., Vergeldt T.F., IntHout J., Kluivers K.B. (2022). Risk factors for primary pelvic organ prolapse and prolapse recurrence: An updated systematic review and meta-analysis. Am. J. Obstet. Gynecol..

[15] Maher C., Feiner B., Baessler K., Christmann-Schmid C., Haya N., Brown J. (2016). Surgery for women with anterior compartment prolapse. Cochrane Database Syst. Rev..

[16] Wu Y.M., Welk B. (2019). Revisiting current treatment options for stress urinary incontinence and pelvic organ prolapse: A contemporary literature review. Res. Rep. Urol..

[17] Billis E., Kritikou S., Konstantinidou E., Fousekis K., Deltsidou A., Sergaki C., Giannitsas K. (2022). The Greek version of the Australian Pelvic Floor Questionnaire: Cross-cultural adaptation and validation amongst women with urinary incontinence. Eur. J. Obstet. Gynecol. Reprod. Biol..

[18] Lin K.Y., Frawley H., Granger C. (2017). Denehy L. The Australian Pelvic Floor Questionnaire is a valid measure of pelvic floor symptoms in patients following surgery for colorectal cancer. Neurourol. Urodyn..

[19] Gleason J.L., Parden A.M., Jauk V., Ballard A., Sung V., Richter H.E. (2015). Outcomes of midurethral sling procedures in women with mixed urinary incontinence. Int. Urogynecology J..

[20] Sanchez-Sanchez B., Torres-Lacomba M., Yuste-Sánchez M.J., Navarro-Brazalez B., Pacheco-da-Costa S., Gutierrez-Ortega C., Zapico-Goni A. (2013). Cultural adaptation and validation of the Pelvic Floor Distress Inventory short form (PFDI-20) and Pelvic Floor Impact Questionnaire short form (PFIQ-7) Spanish versions. Eur. J. Obstet. Gynecol. Reprod. Biol..

[21] Bochenska K., Grzybowska M.E., Piaskowska-Cala J., Mueller M., Lewicky-Gaupp C., Wydra D., Kenton K. (2021). Translation and validation of the polish version of the pelvic floor impact questionnaire short form 7. Int. Urogynecol. J..

[22] Parvathavarthini K.V., Vanusha A. (2018). Clinical epidemiological study of uterine prolapse. Int. J. Reprod. Contracept. Obstet. Gynecol..

[23] Joseph N., Krishnan C., Reddy B.A., Adnan N.A., Han L.M., Min Y.J. (2016). Clinical profile of uterine prolapse cases in South India. J. Obstet. Gynaecol. India.

[24] Gumanga S.K., Munkaila A., Malechi H. (2014). Social demographic characteristics of women with pelvic organ prolapse at the Tamale Teaching Hospital, Ghana. Ghana Med. J..

[25] Yuk J.S., Lee J.H., Hur J.Y., Shin J.H. (2018). The prevalence and treatment pattern of clinically diagnosed pelvic organ prolapse: A Korean National Health Insurance Database-based cross-sectional study 2009–2015. Sci. Rep..

[26] Al-Badr A., Saleem Z., Kaddour O., Almosaieed B., Dawood A., Al-Tannir M., AlTurki F., Alharbi R., Alsanea N. (2022). Prevalence of pelvic floor dysfunction: A Saudi national survey. BMC Womens Health.

[27] Malaekah H., Al Medbel H.S., Al Mowallad S., Al Asiri Z., Albadrani A., Abdullah H. (2022). Prevalence of pelvic floor dysfunction in women in Riyadh, Kingdom of Saudi Arabia: A cross-sectional study. Womens Health.

[28] Mekonnen B.D. (2020). Prevalence and factors associated with uterine prolapse among gynecologic patients at university of Gondar comprehensive specialized hospital, northwest Ethiopia. J. Womens Health Care.

[29] Lin Y.L., Lo T.S., Long C.Y., Law K.S., Ho C.H., Wu M.P. (2020). Time-frame comparison of hystero-preservation in the surgical treatment of uterine prolapse: A population-based nation-wide follow-up descriptive study, 2006–2013 versus 1997–2005. Int. Urogynecol. J..

[30] Weintraub A.Y., Glinter H., Marcus-Braun N. (2020). Narrative review of the epidemiology, diagnosis and pathophysiology of pelvic organ prolapse. Int. Braz J. Urol..

[31] Kayembe A.T., Kayembe C.D.K., Bebele J.P.K., Tozin R.R. (2021). Factors associated with genital prolapse to Saint Joseph Hospital of Kinshasa. Pan. Afr. Med. J..

[32] Wu J.M., Vaughan C.P., Goode P.S., Redden D.T., Burgio K.L., Richter H.E., Markland A.D. (2014). Prevalence and trends of symptomatic pelvic floor disorders in U.S. women. Obstet. Gynecol..

[33] Zeleke B., Bell R., Billah B., Davis S. (2016). Symptomatic pelvic floor disorders in community-dwelling older Australian women. Maturitas.

[34] Tegerstedt G., Maehle-Schmidt M., Nyrén O., Hammarström M. (2005). Prevalence of symptomatic pelvic organ prolapse in a Swedish population. Int. Urogynecol. J. Pelvic. Floor Dysfunct..

[35] Islam R.M., Bell R.J., Billah B., Hossain M.B., Davis S.R. (2016). The prevalence of symptomatic pelvic floor disorders in women in Bangladesh. Climacteric.

[36] Tugume R., Lugobe H.M., Kato P.K., Kajabwangu R., Kanyesigye H., Masembe S., Kayondo M. (2022). Pelvic organ prolapse and its associated factors among women attending the gynecology outpatient clinic at a tertiary hospital in southwestern Uganda. Int. J. Womens Health.

[37] Handa V.L., Blomquist J.L., Knoepp L.R., Hoskey K.A., McDermott K.C., Muñoz A. (2011). Pelvic floor disorders 5–10 years after vaginal or cesarean childbirth. Obstet. Gynecol..

[38] Awotunde O.T., Fehintola A.O., Ogunlaja O.A., Olujide L.O., Aaron O.I., Bakare B., Ogunlaja I.P. (2016). An audit of uterovaginal prolapse in Ogbomoso, south-west Nigeria. Res. J. Health Sci..

[39] Saimin J., Hafizah I., Indriyani N., Wicaksono S. (2020). Uterine prolapse in postmenopausal women in the coastal areas. Indones. J. Obstet. Gynecol..

[40] Metz M., Junginger B., Henrich W., Baeßler K. (2017). Development and validation of a questionnaire for the assessment of pelvic floor disorders and their risk factors during pregnancy and postpartum Geburtshilfe Frauenheilkd. Geburtshilfe Frauenheilkd..

[41] Shrestha B., Onta S., Choulagai B., Paudel R., Petzold M., Krettek A. (2015). Uterine prolapse and its impact on quality of life in the Jhaukhel-Duwakot Health Demographic Surveillance Site, Bhaktapur, Nepal. Glob. Health Action.

[42] Baessler K., O’Neill S., Maher C., Battistutta D. (2009). Australian pelvic floor questionnaire: A validated interviewer-administered pelvic floor questionnaire for routine clinic and research. Int. Urogynecol. J. Pelvic. Floor Dysfunct..

